# The Importance of Formalizing Computational Models of Face Adaptation Aftereffects

**DOI:** 10.3389/fpsyg.2016.00815

**Published:** 2016-06-13

**Authors:** David A. Ross, Thomas J. Palmeri

**Affiliations:** ^1^Department of Psychological and Brain Sciences, University of MassachusettsAmherst, MA, USA; ^2^Department of Psychology, Vanderbilt UniversityNashville, TN, USA

**Keywords:** face recognition, adaptation, exemplar, norm, computational modeling

## Abstract

Face adaptation is widely used as a means to probe the neural representations that support face recognition. While the theories that relate face adaptation to behavioral aftereffects may seem conceptually simple, our work has shown that testing computational instantiations of these theories can lead to unexpected results. Instantiating a model of face adaptation not only requires specifying how faces are represented and how adaptation shapes those representations but also specifying how decisions are made, translating hidden representational states into observed responses. Considering the high-dimensionality of face representations, the parallel activation of multiple representations, and the non-linearity of activation functions and decision mechanisms, intuitions alone are unlikely to succeed. If the goal is to understand mechanism, not simply to examine the boundaries of a behavioral phenomenon or correlate behavior with brain activity, then formal computational modeling must be a component of theory testing. To illustrate, we highlight our recent computational modeling of face adaptation aftereffects and discuss how models can be used to understand the mechanisms by which faces are recognized.

## Introduction

Adaptation aftereffects have been used as a tool for studying low-level vision (e.g., Gibson and Radner, 1937; Blakemore and Sutton, 1969; Frisby, 1979; Wade, 1994; Anstis et al., 1998; Kohn, 2007; Thompson and Burr, 2009; Webster, 2015) and there has been of growing interest in their potential for informing high-level vision. Like low-level aftereffects (color and tilt) just a few seconds of adaptation are needed to bias the perception of high-level stimuli (faces, bodies, and objects; Leopold et al., 2005; Rhodes et al., 2007; Javadi and Wee, 2012; Rhodes et al., 2013). For example, brief adaptation to a face with a wide jaw and narrow eye separation will cause an aftereffect, biasing perception toward a psychologically opposite stimulus, such that a subsequently presented face may appear to have a narrower jaw and wider eye separation (Leopold et al., 2001; Rhodes and Jeffery, 2006; Robbins et al., 2007; Tangen et al., 2011).

The face recognition literature has used adaptation to address debates about the nature of face representations. Unlike low-level visual features, such as color or orientation, faces are complex with high dimensionality. The concept of *face space* has pervaded theorizing about face recognition, providing a framework for understanding how such multidimensional stimuli are represented in the brain (Valentine, 1991). Faces are represented as points along a collection of psychological dimensions, with the distance between points reflecting the similarity between faces. Each dimension of face space is assumed to represent some component of variation across the population of know faces; dimensions could correspond to physical differences, such as the distances between the eyes, or more holistic sources (O’Toole et al., 1993; Burton et al., 1999; Dailey and Cottrell, 1999; Wilson et al., 2002).

Debate has centered on the nature of the face representations that reside within face space (see Valentine, 2001; Rhodes et al., 2005; Rhodes and Leopold, 2011; Valentine et al., 2015). Norm models propose that faces are represented with respect to their deviation from a prototypical or average face, the *norm* (Valentine, 1991; Byatt and Rhodes, 1998; Loﬄer et al., 2005; Rhodes and Jeffery, 2006), whereas exemplar models propose that faces are represented with respect to their similarity to individually encoded faces, *exemplars* (Valentine, 1991; Lewis, 2004).

Although representations assumed by norm and exemplar models are fundamentally different, both turn out to make similar predictions about key aspects of face recognition, such as effects of typicality and distinctiveness (e.g., Valentine and Bruce, 1986; Valentine, 1991) and effects of caricature (e.g., Lewis and Johnston, 1998, 1999; Lewis, 2004). One seemingly promising approach to differentiating predictions of norm and exemplar models relies on face adaptation (e.g., Leopold et al., 2001; Rhodes and Jeffery, 2006; Robbins et al., 2007).

## Face Adaptation Aftereffects

**FIGURE 1:**
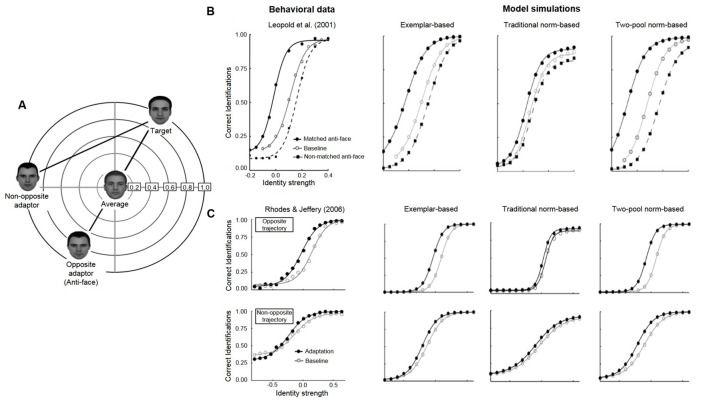
**(A)** Schematic face-space representation of the stimuli used in anti-face adaptation paradigms (adapted from Rhodes and Jeffery, 2006). In both Leopold et al. (2001) and Rhodes and Jeffery (2006), an opposite adaptor (anti-face) was constructed to lie on the opposite side of the norm from each of four target faces (only one shown here), with the opposite test trajectory lying in between. In addition, Rhodes and Jeffery (2006) included additional four non-opposite (control) trajectories (only one trajectory is shown here) that lay between each target face and a corresponding non-opposite adaptor (matched to the opposite adaptor for distance from the target). **(B)** Sensitivity to face identity in baseline (open circles), matched anti-face (closed circles) and non-matched anti-face (open circles) conditions. The proportion of correct responses has been averaged across the four identity trajectories and a best-fitting four parameter logistic function is shown for each condition. Left panel: behavioral data from Leopold et al. (2001). Right panel: simulated data from Ross et al. (2014) for the exemplar-based (left), traditional norm-based (center), and two-pool norm-based (right) models. **(C)** Sensitivity to face identity on baseline (open circles) and adaptation (closed circles) trials along both the opposite morph-trajectory (top) and non-opposite morph trajectory (bottom). Left panel: behavioral data from Rhodes and Jeffery (2006). Right panel: simulated data from Ross et al. (2014) for the exemplar-based (left), traditional norm-based (center), and two-pool norm-based (right) models. Copyright (2013) Psychonomic Society, reproduced from Ross et al. (2014) with permission from Springer Science & Business Media and the Psychonomic Society.

In a now classic paper, Leopold et al. (2001) had participants first learn to identify four novel target faces (Adam, Jim, John, and Henry) and then tested their ability to identify test faces as one of the four studied targets. Test faces were positioned along morph lines that projected from each of the target identities, through the norm, to the opposite side of face space (**Figure 1**). On some trials, participants were first briefly shown an adaptor face for a few seconds and then identified a test face. Without adaptation, the average face at the center of the space was equally likely to be identified as one of the four targets. But with exposure to an adaptor located on the opposite side of the norm from a target, call it “anti-Adam,” the average face was more likely identified as “Adam.” Relative to baseline without adaptation, the psychometric function for identification as a function of the distance of a test face from the average (identity strength), is significantly shifted to the left by adaptation to a matching anti-face (adaptation to anti-Adam, testing on morphs of Adam); this contrasts with the psychometric function for non-matching anti-faces (adaptation to anti-Jim, testing on morphs of Adam), which is slightly shifted to the right.

Rhodes and Jeffery (2006) extended this paradigm with the inclusion of a critical control condition. In addition to contrasting adaptation along opposite morph trajectories that passed through the norm (Leopold et al., 2001), they tested adaptation along non-opposite morph trajectories that did not (**Figure 1**). Adaptation affected face identification with respect to the norm, such that post-adaptation identification thresholds were significantly lower for faces along opposite trajectories, compared to those along non-opposite trajectories. Rhodes and Jeffery (2006) reasoned that since the magnitude of adaptation was dependent on whether the morph trajectory passed through the norm (opposite trajectories) or not (non-opposite trajectories), the psychological representation of faces must make some reference to a norm face.

## Intuitions About Face Adaptation

Finding that adaptation biases face recognition with respect to the norm rather than simply biasing recognition away from the adaptor has been widely interpreted as evidence for norm-based coding (Rhodes et al., 2005; Rhodes and Jeffery, 2006; Robbins et al., 2007; Susilo et al., 2010; Rhodes and Leopold, 2011; Rhodes and Calder, 2014; Short et al., 2014; Walsh et al., 2015, but see Zhao et al., 2011; Storrs and Arnold, 2012). At its most simplest, finding that face adaptation is sensitive to the norm intuitively suggests that face representations are constructed with respect to a norm.

**FIGURE 2:**
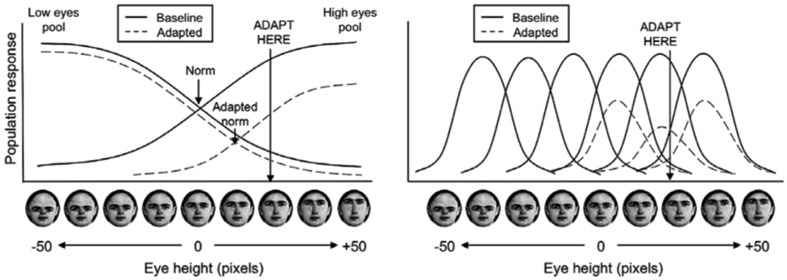
**Illustration of intuitive, hypothesized adaptation effects from a two-pool model (left) and exemplar-based model (right) reproduced from Susilo et al. (2010).** The dimension used in this illustration is eye height. In the two-pool model **(left)**, eye height is encoded by opposing pools of face representations, centered on the norm with the combined activity of the pools representing the location of a face on the eye height dimension. In the exemplar-based model **(right)**, eye-height is encoded by multiple representations with bell-shaped tunings. In both cases, adaptation is assumed to result in a decrease in activation of each representation in proportion to its activation by the adapting stimulus. The effect of adaptation of the face representations is represented by the dashed line. Simple illustrations such as these often drive theoretical intuitions about face adaptation aftereffects. Copyright (2010) Association for Research in Vision and Ophthalmology (RVO).

Intuitions are also often supported by considering illustrations of one-dimensional two-pool (norm) and multichannel (exemplar) models (**Figure 2**). In a two-pool model, face representation are assumed to be broadly tuned, responding maximally to a particular extreme within face space. For example, in **Figure 2**, the two pools encode variations in eye height, with one pool preferring faces with extremely high eyes and the other pool preferring faces with extremely low eyes. The location of a face along a given dimension is encoded by the proportional activity of the two opposing pools. Because the pools are explicitly specified to intersect at the location of the norm face, two-pool models are generally considered instantiations of norm models. By contrast, in a multichannel model, face representations are assumed to be narrowly tuned, preferring faces at a particular location along some dimension. These are generally considered analogous to exemplar models because faces are encoded only with respect to other faces, rather than making reference to any norm face.

For both two-pool and multichannel models, face representations are assumed to adapt in proportion to their level of activation to the adaptor. In the case of the two-pool norm model, adapting one of the pools more strongly than the other will cause a subsequent bias in the relative activation of the two pools, with the effect that after adaptation, perception will be biased toward the opposite extreme. By contrast, for the multichannel exemplar models, adaptation will only affect representations at the location of the adaptor, causing subsequent perception to be biased away from the adaptor, not specifically toward the opposite extreme.

A significant limitation of this past work is that nearly all of the assumptions about how norm and exemplar models might respond to face adaptation have been based on intuitions and pictorial illustrations of one- or two-dimensions. Rarely are these intuitions supported by explicit simulations, where predictions are formally generated, compared, and evaluated. The mechanisms involving face recognition undoubtedly involves combinations of high-dimensionality, non-linear activation, and parallelism – all properties of the human brain. Making predictions based on intuition or illustration alone must be the subject of scrutiny (e.g., see Hintzman, 1990; Burton and Vokey, 1998; Lewis, 2004; Palmeri and Cottrell, 2009), as we recently illustrated (Ross et al., 2014).

## Modeling Face Adaptation Aftereffects

Part of our motivation for a close examination of formal predictions of norm and exemplar models comes from lessons learned in the categorization literature. Posner and Keele (1968, 1970) had people learn to classify novel dot pattern stimuli into different categories, each defined by a unique prototype. Even though they never saw the prototypes during learning, being trained only on distortions of prototypes, when tested after learning, classification of unseen prototypes was better than classification of distortions. The canonical interpretation of prototype enhancement (e.g., Reed, 1972; Rosch, 1975; Homa, 1984) was that category learning involves some form of prototype abstraction. How else could an unseen prototype be classified so well than it had been abstracted as a product of learning? These intuitions were challenged when prototype and exemplar models were formalized in mathematics and simulations and their predictions explicitly generated and compared to data. Models that assume memory for category exemplars, without any abstraction, account quite well for prototype enhancement effects as well as phenomena that pure prototype abstraction models cannot (e.g., see Busemeyer et al., 1984; Hintzman, 1986; Nosofsky, 1992; Palmeri and Nosofsky, 2001). Could a similar disconnect between intuition and formal prediction be the case for norm versus exemplar debate in face recognition?

Ross et al. (2014), we instantiated an exemplar model of face recognition, which bore similarities to exemplar models of categorization (e.g., Nosofsky, 1986; Kruschke, 1992) and descriptions of multichannel models (e.g., Robbins et al., 2007). We also instantiated two versions of a norm-based model. One version, which we referred to as a traditional norm-based model, had been formalized previously by Giese and Leopold (2005); the other, a two-pool model, was adapted from descriptions of norm-based coding (e.g., Rhodes and Jeffery, 2006). In the following descriptions, we omit many of the mathematical and computational details, focusing instead on what we did and why, and recommend the reader to refer to Ross et al. (2014).

Our core modeling framework is no different from dozens, if not hundreds, of other models of perception and cognition. There is an input layer that models the perceptual representation of the face, an intermediate layer that models alternative assumptions about face space (exemplar, traditional norm, and two-pool), and an output layer that generates a decision about the face (Adam, Jim, John, or Henry). Because we were interested in directly comparing three alternative theories of face space, we assumed the same input representation and output mechanism for every model. All that varied was the internal face-space representation.

To briefly outline, when a test face, say a particular face along the Anti-Adam/Adam morph continuum, is presented to the model for identification, a multidimensional perceptual representation is created by the visual system. Instead of attempting to model the complete processing hierarchy of the ventral stream visual system that creates this perceptual representation (e.g., Jiang et al., 2006; Serre et al., 2007), we considered two simpler possibilities. The first made no specific assumptions about how the perceptual representation of a face is created from its 2D retinal image, and simply assumed that a randomly sampled face is represented by a random sample from a multivariate Gaussian (normal) distribution (see also Lewis, 2004). The second used an actual 2D face image as input and created a multidimensional input representation of it via principal components analysis (PCA), much like many other models (e.g., O’Toole et al., 1993; Burton et al., 1999; Dailey and Cottrell, 1999; Giese and Leopold, 2005; Richler et al., 2007).

In both cases the perceptual representation is simply a vector specifying the location of a given test face along each dimension of face space. This face representation then activates exemplars, norms, or pools in the face-space layer according to the rules for that particular model of face space. In each case, we assumed that pre-experimental experience populated face space with a collection of exemplars, norms, or pools, depending on the model. Like other exemplar-based face-space model (e.g., Lewis, 2004; Giese and Leopold, 2005), activation of a given exemplar in face space is a non-linearly decreasing function of the distance of that exemplar to the test face. For the traditional norm-based model (Giese and Leopold, 2005), norm representations are activated by a test face as a function of both the difference in angular distance with respect to the norm as well as their relative distance from the norm. And for the two-pool model (e.g., Rhodes and Jeffery, 2006), competing pools of units on opposite side of the norm are activated as a function of the relative similarity of a test face to members of each pair in the pool. Simple assumptions were made to implement adaptation within the three models as a temporary rescaling of maximal activation according to the similarity of each exemplar, norm, or pool to the adaptor.

Finally, the distributed pattern of activity across these exemplars, norms, or pools is associated with output nodes for Adam, Jim, John, or Henry, with the relationship between a given pattern of face-space activations and a particular identity learned using a standard delta-rule learning algorithm. This step mirrors the initial learning of face identities by participants in the face adaptation experiments. Activation of the identity nodes is readily translated into identification probabilities (following, for example, Kruschke, 1992). While the actual identification of the learned face is a critical component of face adaptation experiments, most predictions described for different face-space models focus entirely on the face-space representation, largely ignoring how the activation of those representations in face space might be combined to generate the learned face identification response that is the key measure in the task.

We tested the three models on how well they could qualitatively predict the observed data from Leopold et al. (2001) and Rhodes and Jeffery (2006), outlined earlier, as well as a related data from Leopold and Bondar (2005). Each of the variants has a small collection of free parameters, including such things as the number of dimensions in the input perceptual representations, the width of the tuning of exemplars, norms, or pools, and scaling parameters that define things like the strength of adaptation and how output activity is mapped onto response probabilities. We explored a wide range of model parameterizations, and not only looked for parameter combinations that provided a good quantitative account of the observed data in each experiment, but also evaluated whether models made parameter-free qualitative predictions irrespective of particular parameterizations. Simply put, outside of subsets of parameters that produced no significant effect of adaptation at all – for example, having the scaling on adaptation too low or the width of the tuning function too small – in all cases, the quantitative predictions illustrated below map onto the qualitative predictions across parameters sets.

All three models are able to account for the data from Leopold et al. (2001) and only the exemplar model and the two-pool model are able to account for the data from Rhodes and Jeffery (2006), with the traditional norm-based model failing on that score (**Figure 1**). Despite prevalent intuitions that face adaptation aftereffects clearly support norm models over exemplar models, those intuitions were not borne out when those models were explicitly implemented and simulated and their predictions compared to observed data. In fact, our variant of the traditional norm-based model could not explain key aspects of the observed face adaptation data.

## Why Intuitions Fail

Why do common intuitions about norm and exemplar face adaptation fail? To begin with, most illustrations depict one or two dimensions but face space likely has 10, 20, 100, or more dimensions. Human intuition beyond two or three dimensions often fails miserably (e.g., DiCarlo and Cox, 2007). Combine this high dimensionality with the non-linearities in activation functions and decision rules, and intuition is bound to fail.

Consider also the claim that representations in a multichannel model must be narrowly tuned (e.g., Robbins et al., 2007). On a single dimension perhaps this corresponds to a representation having at most significant activation across one quarter of the span of the dimension (**Figure 2**, right). But, consider instead a representation in two dimensions; we now have a circle that occupies significantly less than one quarter (one quadrant) of the total space. While in one dimensions it seems that each exemplar must be so broadly tuned that it would convey little useful information to discriminate different faces, in multiple dimensions this need not be the case.

Illustrations of exemplar models (**Figure 2**, right) also tend to impose a complete tiling across face space. To begin with, while a complete tiling might be feasible for one or even two dimensions, the sheer number of nodes necessary to fully tile a 100-dimension face space is far more than the number of neurons in the brain (and likely more than the number of atoms in the known universe). More importantly, exemplar models assume that face space will be populated based on specific experience with faces, and do not assume any *a priori* covering map across space. The combination of a finite number of face representations, together with non-uniform exemplar distribution, along with exemplar tuning widths required in high dimensions, creates a situation in which face adaptation is dependent on the adaptor faces location relative to the entire population of exemplars. Like other examples from the face recognition and category learning literatures (e.g., Lewis, 2004), the ability of the exemplar model to make predictions that appear to require a norm is driven by the fact that the average is implicitly represented in the statistical distribution of faces, not any explicit norm representation.

## Discussion

Face adaptation aftereffects are used to draw mechanistic conclusions about how faces are represented. Our work makes clear that mechanistic conclusions really must be supported by formalized computational models that delineate specific testable assumptions about how faces are represented, how face knowledge is represented and used, how adaptation works, and how face identities are determined. Our assumptions about any of these components may be wrong, or there may be alternatives that we did not consider. But that is a strength of formal modeling, not a weakness, especially when compared to mechanistic predictions derived from intuitions or simplified illustrations, as has been the case for much theoretical work concerning face adaptation. In nearly every area of perception and cognition where a computational modeling has been deployed, there are examples of empirical phenomena that intuitively point to one particular mechanistic explanation but in fact can be explained as well or better by other explanations when those alternatives are formally evaluated (e.g., Farrell and Lewandowsky, 2010).

## Author Contributions

Both DR and TP contributed equally to the conception, design and drafting of this manuscript and are accountable for all aspects of the work in ensuring that questions related to the accuracy or integrity of any part of the work are appropriately investigated and resolved.

## Conflict of Interest Statement

The authors declare that the research was conducted in the absence of any commercial or financial relationships that could be construed as a potential conflict of interest.
